# The *Escherichia coli* BtuE Protein Functions as a Resistance Determinant against Reactive Oxygen Species

**DOI:** 10.1371/journal.pone.0015979

**Published:** 2011-01-10

**Authors:** Felipe A. Arenas, Paulo C. Covarrubias, Juan M. Sandoval, José M. Pérez-Donoso, James A. Imlay, Claudio C. Vásquez

**Affiliations:** 1 Departamento de Biología, Facultad de Química y Biología, Universidad de Santiago de Chile, Santiago, Chile; 2 Department of Microbiology, University of Illinois, Urbana-Champaign, Illinois, United States of America; Auburn University, United States of America

## Abstract

This work shows that the recently described *Escherichia coli* BtuE peroxidase protects the bacterium against oxidative stress that is generated by tellurite and by other reactive oxygen species elicitors (ROS). Cells lacking *btuE* (Δ*btuE*) displayed higher sensitivity to K_2_TeO_3_ and other oxidative stress-generating agents than did the isogenic, parental, wild-type strain. They also exhibited increased levels of cytoplasmic reactive oxygen species, oxidized proteins, thiobarbituric acid reactive substances, and lipoperoxides. *E. coli* Δ*btuE* that was exposed to tellurite or H_2_O_2_ did not show growth changes relative to wild type cells either in aerobic or anaerobic conditions. Nevertheless, the elimination of *btuE* from cells deficient in catalases/peroxidases (Hpx^−^) resulted in impaired growth and resistance to these toxicants only in aerobic conditions, suggesting that BtuE is involved in the defense against oxidative damage. Genetic complementation of *E. coli* Δ*btuE* restored toxicant resistance to levels exhibited by the wild type strain. As expected, *btuE* overexpression resulted in decreased amounts of oxidative damage products as well as in lower transcriptional levels of the oxidative stress-induced genes *ibpA*, *soxS* and *katG*.

## Introduction

Although the tellurium oxyanion, tellurite (TeO_3_^2−^), is toxic to most microorganisms, the ultimate basis of its toxicity has remained elusive. Available evidence from *Escherichia coli* [1], *Pseudomonas pseudoalcaligenes* KF707 [2] and *Rhodobacter capsulatus* [3] supports the idea that bacterial tellurite toxicity is related to oxidative stress. In particular, *E. coli* exposed to K_2_TeO_3_ exhibits increased levels of cytoplasmic reactive oxygen species (ROS), mainly superoxide (O_2_^−^) [4]. In turn, increased O_2_^−^ levels can trigger a number of metabolic effects, including protein and membrane oxidation, induction of antioxidant enzymes and inactivation of [4Fe-4S] clusters from certain dehydratases [5]–[9].

Aerobic organisms protect themselves from ROS by synthesizing antioxidant enzymes as well as low molecular weight molecules such as ascorbate and glutathione [7], [10]. *E. coli *contains several antioxidant enzymes, including catalases (*katG and katE*) [11]–[13], superoxide dismutases (MnSOD, FeSOD, and CuZnSOD) [13]–[15], alkylhydroperoxidase [13] and thiol peroxidase [16]. To cope with oxidative stress, the genes encoding these enzymes are often induced by ROS, whether it is produced in different compartments of the bacterial cell or at different growth stages [13].

Glutathione peroxidases (GPXs) are another kind of antioxidant enzyme that in eukaryotes plays an important role in defending the cell against hydroperoxides and lipid peroxides [17], [18]. Conversely, in prokaryotes the available information about GPXs is still very limited. However, a recent report identified and characterized the Se-independent GPX BtuE from *E. coli*, which *in vitro* can catalyze the decomposition of a variety of peroxides, mainly lipid peroxides, using thioredoxins A or C as the reducing agent. It was also shown that, like other *E. coli* antioxidant genes, *btuE* is induced under oxidative stress conditions [19].

Tellurite toxicity is due at least in part to the generation of oxidative stress that alters different cellular processes [9]; therefore, the role of the *E. coli btuE* gene product was examined *in vivo*. The *btuE* gene was cloned and its effects were analyzed in cells exposed to various ROS elicitors. Results were compared to those obtained with mutants lacking *btuE* and to genetically complemented Δ*btuE* cells. Taken together, the emerging picture is that BtuE is involved in protecting the cell from the deleterious effects caused by exposure to tellurite as well as to other ROS elicitors.

## Results

### BtuE mediates resistance to ROS elicitors in *E. coli*

To assess whether BtuE plays a role in the resistance of *E. coli* to oxidative stress, growth inhibition zones were determined for wild-type, *btuE-*overexpressing (pBAD/*btuE*), *btuE*-deficient (Δ*btuE*) and genetically complemented *btuE* mutant (Δ*btuE* pBAD/*btuE*) cells (Table 1). Tested ROS elicitors included the superoxide-generating potassium tellurite [4], the hydroxyl radical elicitor chromate [20], [21], and hydrogen peroxide [7]. Cadmium chloride, whose toxicity seems not to involve ROS generation, was used as control [22].

**Table 1:** BtuE mediates resistance to ROS elicitors in *E. coli*.

	Growth inhibition zone (cm^2^)			
Strain	K_2_TeO_3_	H_2_O_2_	K_2_CrO_4_	CdCl_2_
BW25113 pBAD	6.7±0.3	5.7±0.1	6.44	4.2±0.2
BW25113 pBAD/*btuE*	5.0±0.3	3.3±0.1	3.4±0.1	4.0±0.1
Δ*btuE* pBAD	8.1±0.1	6.7±0.1	7.4±0.1	4.2±0.1
Δ*btuE* pBAD/*btuE*	4.6±0.2	3.5±0.2	4.1±0.1	3.8±0.2

Growth inhibition zones for wild type, *btuE-*overexpressing (pBAD/*btuE*), *btuE*-deficient (Δ*btuE*), and genetically complemented *btuE* mutant (Δ*btuE* pBAD/*btuE*) cells were determined as described in Methods. Cells growing in the presence of 0.2% arabinose were exposed to K_2_TeO_3_ (10 µl, 1 mg ml^−1^), H_2_O_2_ (10 µl, 3% v/v), K_2_CrO_4_ (10 µl, 1 M) and CdCl_2_ (10 µl, 1 M). Parentheses indicate the amount and concentration of each toxin that was applied to the disks. Values are the mean of 4 to 6 independent experiments ± SD.

Cells overexpressing *btuE* exhibited increased resistance to compounds whose toxicity involves ROS generation. Conversely, the Δ*btuE* strain showed increased sensitivity to all these compounds relative to wild type controls. Genetically complemented Δ*btuE* cells exhibited resistance levels to K_2_TeO_3_, K_2_CrO_4_ and H_2_O_2_ that were nearly identical to those observed for the *btuE*-overexpressing wild type strain. In contrast, all tested strains showed similar sensitivity to the non-ROS-producer, thiol oxidizer, CdCl_2_ (Table 1).

Interestingly, when minimal inhibitory concentrations (MIC) were determined in liquid medium, the H_2_O_2_ MIC for pBAD/*btuE* cells was ten-fold higher than that of the parental, isogenic, control strain (Table S2). This result supports the previous observation that BtuE can function as a glutathione peroxidase *in vitro* [19].

### BtuE protects *E. coli* from intracellular ROS

Cytoplasmic ROS levels were assessed using the probe 2′,7′-dihydrodichlorofluorescein diacetate, as described in Methods. All strains exposed to K_2_TeO_3_, paraquat or K_2_CrO_4_ exhibited significant probe activation; the slight probe activation observed in untreated cells is presumed to be related to metabolic ROS generation. In the absence of exogenous oxidants, mutants lacking *btuE* showed higher ROS content than did wild type cells. The *E. coli* pBAD/*btuE* strain and the complemented Δ*btuE* mutants showed decreased levels of probe activation relative to non-overproducing strains (Table 2).

**Table 2:** *btuE* expression results in decreased intracellular ROS.

	Fluorescence (AU/mg protein×10^3^)			
*E. coli strain*	Control	K_2_TeO_3_	Paraquat	K_2_CrO_4_
BW25113 pBAD	14.6±0.8	26.5±0.8	22.0±1.7	96.1±3.0
BW25113 pBAD/*btuE*	10.0±1.2	20.0±1.2	12.7±2.2	47.2±2.8
Δ*btuE* pBAD	19.1±2.2	26.5±0.8	23.1±0.9	95.0±3.1
Δ*btuE* pBAD/*btuE*	10.1±1.0	20.8±0.7	13.4±2.4	48.3±1.8

Cytoplasmic ROS content was assessed by measuring the activation of 2′,7′-dihydrodichlorofluorescein diacetate in wild type, pBAD/*btuE*, Δ*btuE* and Δ*btuE* pBAD/*btuE* cells as described in Methods. Cells were induced with 0.2% arabinose and exposed to K_2_TeO_3_ (0.5 µg ml^−1^), paraquat (50 µg ml^−1^) or K_2_CrO_4_ (1 mM) for 15 min at 37°C. Fluorescence (AU, arbitrary units) was determined and normalized per mg of protein. Values represent the mean of three independent trials ± SD.

To further analyze the protective role of BtuE against ROS generated during the normal metabolism, we studied the effect of overexpressing *btuE* in strains lacking superoxide dismutases (Δ*sodAB*) or catalases/peroxidases (Hpx^−^). These strains suffer increased levels of O_2_^−^ and H_2_O_2_, respectively [23], [24]. Superoxide as well as peroxide levels were assessed by flow cytometry as described in Methods. BtuE production resulted in decreased ROS levels, showing a protective effect both in basal metabolic conditions as well as during oxidative stress caused by ROS elicitors (Fig. S1).

### BtuE production results in decreased protein oxidation and damage to membrane lipids

The formation of carbonyl groups in some amino acid side chains is a conventional marker of ROS-mediated protein oxidation [25]. Spectrophotometric determination of derivatized carbonyl groups with 2,4-dinitrophenylhydrazine showed that *E. coli* Δ*btuE* exhibited increased protein oxidation -even in the absence of toxicants- as compared to wild type cells. Genetic complementation of *E. coli* Δ*btuE*, as well as overexpression of *btuE*, resulted in decreased protein oxidation, regardless of the ROS elicitor (Table 3).

**Table 3:** *btuE* expression alleviates oxidation of cytoplasmic proteins.

	Carbonyl groups (µmol/mg protein)		
*E. coli* strain	Control	K_2_TeO_3_	H_2_O_2_
BW25113 pBAD	8.1±5.2	25.0±3.1	16.7±1.2
BW25113 pBAD/*btuE*	9.6±2.9	10.3±0.9	12.3±2.2
Δ*btuE* pBAD	17.1±6.0	33.0±10.1	39.6±5.0
Δ*btuE* pBAD/*btuE*	8.3±0.9	9.6±1.4	13.2±4.6

Protein oxidation was determined in wild type, pBAD/*btuE*, Δ*btuE* and Δ*btuE* pBAD/*btuE* cells by the chemical protein carbonyl assay described in Methods. Total protein present in extracts of cells grown in the presence of 0.2% arabinose and exposed for 30 min to K_2_TeO_3_ (0.5 µg ml^−1^) or H_2_O_2_ (100 µM) were reacted with 2,4-dinitrophenylhydrazine, and the specific carbonyl absorbance was read at 370 nm. Values represent the mean of three independent experiments ± SD.

Thiobarbituric acid responsive substances (TBARS) are routinely used to assess oxidative stress damage to membrane lipids in diverse organisms [26], [27]. TBAR content increased ∼3- and ∼5-fold when *E. coli* was exposed to K_2_TeO_3_ or H_2_O_2_, respectively (Table 4). Even in the absence of toxicant, *E. coli* Δ*btuE* showed increased (∼6-fold) levels of these substances relative to wild-type controls, suggesting that BtuE may function in controlling the level of membrane peroxidation products that are generated during the normal, basal metabolism. Interestingly, thiobarbituric acid responsive substances levels did not increase further when Δ*btuE* cells were exposed to K_2_TeO_3_ or H_2_O_2_ (Table 4).

**Table 4:** Elimination of *btuE* results in increased thiobarbituric acid-reactive substances in *E. coli*.

	pmol TBARS/mg protein		
Strain	Control	K_2_TeO_3_	H_2_O_2_
BW25113	24.6±5.7	81.20±8.0	131.9±18.6
Δ*btuE*	162.0±9.0	147.0±27.1	144.0±2.5

Membrane lipid peroxidation products were determined as thiobarbituric acid-reactive substances (TBARS) in wild type (BW25113) and Δ*btuE* strains in the absence (control) or presence of K_2_TeO_3_ (0.5 µg ml^−1^) or H_2_O_2_ (100 µM) for 30 min. Values represent the mean of three independent experiments ± SD.

Given the above results the level of lipid peroxides was determined in all studied strains, using the method described by Cha et al. [16]. BtuE overproduction resulted in decreased levels of lipid peroxides. Conversely, *btuE*-lacking cells showed increased levels of lipid peroxides regardless of the presence or absence of tellurite or hydrogen peroxide, suggesting that BtuE might participate in preventing membrane damage. As expected, upon genetic complementation *E. coli* Δ*btuE* exhibited decreased levels of lipid peroxides (Table S3).

### *btuE* expression results in decreased induction of *ibpA*, *soxS* and *katG* genes

**Figure 1:**
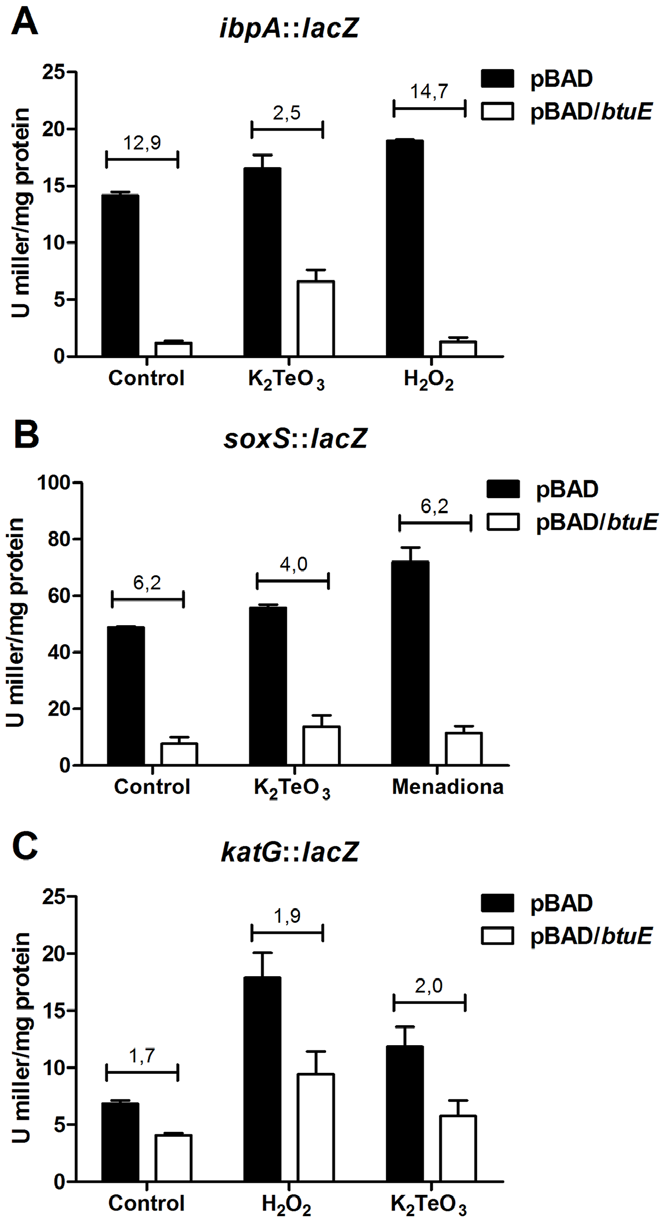
Effect of *btuE* expression on the transcriptional level of *ibpA*, *soxS*, and *katG*. β-galactosidase activity was assayed as described [39] in *E. coli* ADA110 (*ibpA*::*lacZ*) (**A**), SP11 (*soxS*::*lacZ*) (**B**), and GS022 (*katG*::*lacZ*) (**C**) strains carrying pBAD or pBAD/*btuE*. Data are normalized to the concentration of protein. Cells were exposed for 3 h (ADA110), 30 min (SP11) or 25 min (GS022) in the absence (control) or presence of K_2_TeO_3_ (0.5 µg/ml), menadione (100 µM) or H_2_O_2_ (100 µM). Assays were carried out in the presence of 0.2% L-arabinose. Values represent the average of three independent trials ± SD. Numbers above each condition represent the pBAD/pBAD/*btuE* ratio.

The *E. coli* reporter strains ADA110 [4], [28], SP11 and GS022 were used to assess the protective effect that BtuE confers against ROS elicitors. These strains harbor chromosomal insertions of the *lacZ* gene under the control of *ibpA*, *soxS* and *katG* promoters, respectively, which are induced under different stress conditions such as misfolding of cytoplasmic proteins and oxidative stress (*ibpA*), the presence of superoxide-generating compounds (*soxS*), and peroxides (*katG*). The effect of *btuE* overexpression was assessed by transforming them with pBAD/*btuE* or pBAD (control) plasmids and monitoring β-galactosidase activity after exposure to K_2_TeO_3_, menadione or H_2_O_2_. As expected, increased β-galactosidase activity was observed after toxicant exposure for all tested strains under control conditions. In turn, *btuE* overexpression resulted in a considerable decrease of enzyme activity, even in the absence of toxicants (Fig. 1). In fact, by hampering the activation of the *ibpA* promoter in *E. coli* ADA110, *btuE* overexpression resulted in a ∼13- (control), 2.5- (tellurite) and 15-fold (peroxide) diminution of β-galactosidase activity compared to strains harboring the pBAD vector. This result suggests that BtuE might protect the cell by decreasing oxidative stress and cytoplasmic protein misfolding, whether these are generated by basal metabolism or by ROS elicitors (Fig. 1A).

In addition, Figs. 1B and C show that *btuE* overexpression results in >4- (*soxS*) and ∼2-fold (*katG*) decrease in β-galactosidase activity relative to strains harboring pBAD vector only. By diminishing the response of the ROS defense regulons *soxRS* and *oxyR*, these results suggest that BtuE might help to alleviate oxidative stress in the *E. coli* cytoplasm.

### BtuE protects *E. coli* lacking catalases and peroxidases from oxidative stress

**Figure 2:**
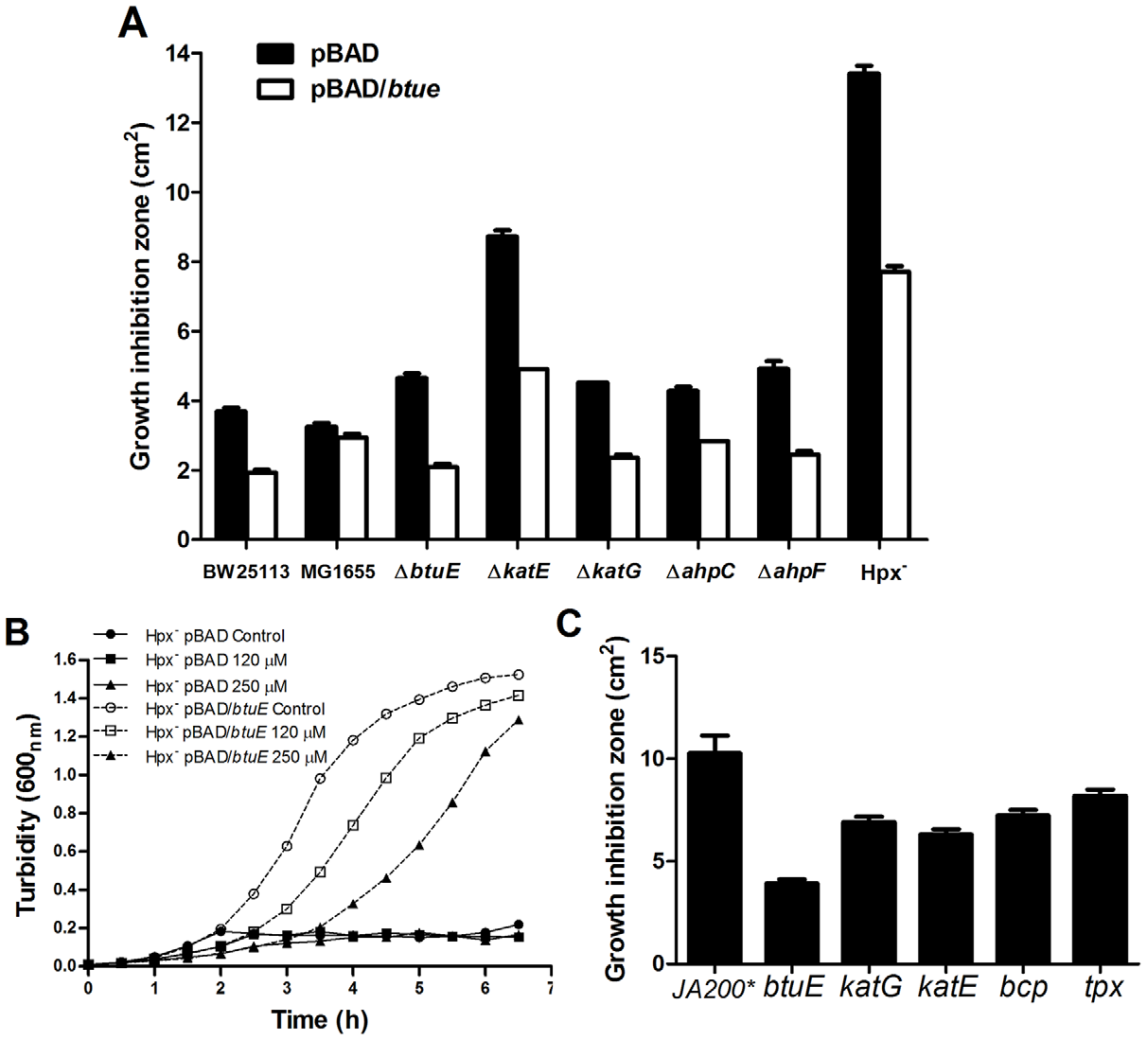
BtuE protects *E. coli* from peroxide damage. **A**, wild-type (BW25113 and MG1655), Δ*btuE*, catalase-deficient (Δ*katG*, Δ*katE*), alkyl hydroperoxidase-deficient (Δ*ahpC*, Δ*ahpF*), and Hpx^−^ cells carrying pBAD or pBAD/*btuE* were grown aerobically in the presence of 0.2% arabinose and exposed to H_2_O_2_ (10 µl, 1 M). Growth inhibition zones represent the mean of three independent experiments ± SD. **B**, growth curves of Hpx^−^ cells carrying the indicated plasmids exposed to 120 or 250 µM hydrogen peroxide. **C**, *E. coli* expressing the indicated peroxidases were grown in the presence of 1 mM IPTG and exposed to H_2_O_2_ (10 µl, 1 M). Parentheses indicates the amount and concentration of H_2_O_2_ that was applied to the disks. Bars represent the average of three independent experiments ± SD. JA200*, parental, isogenic strain that does not overexpress the analyzed peroxidases.

The *btuE* gene was expressed in different *E. coli* genetic backgrounds, and growth inhibition zones were determined. Fig. 2A shows that all strains exhibited greater H_2_O_2_ tolerance when *btuE* was overexpressed. Similar results were obtained for potassium tellurite (not shown). The same trend was observed when growth curves of the Hpx^−^ strain overexpressing *btuE* were analyzed for both H_2_O_2_ (Fig. 2B) or K_2_TeO_3_ (not shown). These data support the idea that the GPX activity of BtuE protects *E. coli* from H_2_O_2_ exposure.

Given the protective effect of BtuE in *E. coli* Hpx^−^ against the tested ROS elicitors, it was of interest to analyze the effect of overexpressing other peroxidase genes in this bacterium. Fig. 2C shows that BtuE generates higher H_2_O_2_ resistance than KatG and KatE catalases or BCP and Tpx peroxidases.

### BtuE production results in increased resistance of *E. coli* to potassium tellurite and hydrogen peroxide only in aerobic conditions

Since BtuE exhibits peroxidase activity *in vitro*, it was reasoned that transferring the Δ*btuE* mutation to an Hpx^−^ genetic background could help in analyzing the net effect of BtuE when other H_2_O_2_ scavenging enzymes are missing. As seen in Fig. S2, the absence of *btuE* rendered Hpx^−^ cells even more sensitive to TeO_3_^2−^ in the presence of oxygen. When growth curves were analyzed, the effect of the *btuE* mutation was more evident in aerobic conditions (Fig. S2A–B). The oxygen requirement was confirmed by determining growth inhibition zones (Fig. S2C).

Finally, the effect of the *btuE* mutation upon H_2_O_2_ tolerance in an Hpx^−^ genetic background was evaluated. Fig. S2D–E shows that growth of Hpx^−^ Δ*btuE* cells is more sensitive to hydrogen peroxide than that of the parental Hpx^−^ strain only in aerobic conditions. Again, these results were confirmed by determining growth inhibition zones (Fig. S2F).

## Discussion

Since heavy metal pollution is a serious problem worldwide, there is a growing need to elucidate its toxic effects in sensitive microorganisms. It is also of interest as to unveil the resistance mechanisms that protect resistant bacteria [29]–[32].

The toxicity of some metals is mediated by the generation of oxidative stress, so the cell must invoke a number of antioxidant defences–both enzymatic and non-enzymatic–to cope with this situation. In this regard, tellurite toxicity was initially thought to arise mainly from its ability to oxidize several cellular components [5], [6], [33]. Later it was recognized that the tellurium oxyanion triggers a series of events that leads to the generation of ROS, particularly superoxide [2], [4], [8], [9], [32], [34].

The dearth of knowledge about prokaryotic glutathione peroxidases prompted us to analyze the role of the *E. coli btuE* gene product in cellular resistance to ROS. To assess if BtuE displays a general antioxidant function *in vivo*, the effect of *btuE* in wild-type, pBAD/*btuE*, Δ*btuE* and Δ*btuE* pBAD/*btuE* cells exposed to potassium tellurite and other ROS elicitors was evaluated. While in general terms Δ*btuE* mutants were more sensitive to ROS elicitors, *btuE* overexpression resulted in enhanced cellular resistance to tellurite (∼4-fold) and hydrogen peroxide (∼10-fold) as compared to parental, wild type cells. Similar results were observed when cells were exposed to chromium, a generator of hydroxyl radicals [20], [21]. In contrast, BtuE did not influence *E. coli* resistance to CdCl_2_ (Table 1, Table S2).

To test whether BtuE might affect the level of intracellular ROS, the fluorescent, oxidation-sensitive probe 2′,7′-dihydrodichlorofluorescein diacetate was used. Tellurite, paraquat or chromate exposure resulted in increased ROS levels, above those observed in unexposed cells. *E. coli* Δ*btuE* always exhibited higher basal ROS levels than wild type cells; conversely, *E. coli* pBAD/*btuE* showed ROS levels far below those observed in controls (Table 2). Similar results were observed when protein carbonylation was assessed (Table 3), suggesting that BtuE could participate in the response to oxidative stress by lowering cytoplasmic ROS levels.

Since thiobarbituric acid responsive substances have been used routinely to assess oxidative stress damage to lipids [4], [26], [27], the effect of BtuE on membrane lipid damage was studied. A high increase (∼6-fold) in the levels of these compounds was observed in *E. coli* Δ*btuE* in the absence of any toxicant, suggesting that BtuE may function in preventing damage to membrane lipids or controlling the level of membrane peroxidation products (Table 4). Given that BtuE exhibits higher peroxidase activity with lipid peroxides *in vitro* [19], the *in vivo* situation was analyzed. Table S3 shows that BtuE is involved specifically in lowering lipid peroxide levels in *E. coli*, again indicating the importance of BtuE in membrane damage. In this context, it is interesting that Se-independent glutathione peroxidases preferentially degrade lipid peroxides [18], [35], [36].

Since tellurite toxicity is highly dependent on the presence of oxygen [1], [37] and GPXs are involved in oxidative stress, the role of BtuE in *E. coli* exposed to K_2_TeO_3_ both in aerobic and anaerobic conditions was analyzed. It was observed that in aerobic conditions the introduction of the *btuE* mutation into an Hpx^−^ background resulted in impaired growth and in increased tellurite sensitivity (Fig. S2). Similar results were observed with hydrogen peroxide, except that in anaerobic conditions *E. coli* wild type strains (BW25113 and MG1655) as well as the Δ*btuE* strain showed higher H_2_O_2_ sensitivity. This may be due to the fact that in aerobic conditions cells display fully induced antioxidant mechanisms to cope with peroxide [7]. In addition, no difference in peroxide resistance was observed between Hpx^−^ and Hpx^−^ Δ*btuE* strains, suggesting that BtuE is important only in aerobic conditions (Fig. S2D–E–F). In support of this, when *btuE* was expressed in *E. coli* defective in H_2_O_2_-scavenging, increased H_2_O_2_ tolerance was observed in all *btuE*-complemented mutants (Fig. 2A–B).

Given that BtuE also efficiently decomposes lipid peroxides *in vitro* [19], we speculate that although adding hydrogen peroxide in anaerobic conditions can trigger a number of oxidative events, lipid peroxidation will not occur since it requires molecular oxygen. In this context, the toxic substrates of BtuE will be missing so that the enzyme will have no effect. Further experiments to unveil the global role of BtuE in the *E. coli* oxidative metabolism are under way in our laboratory.

## Materials and Methods

### Bacterial strains and culture conditions

Bacterial strains used in this study are listed in Table S1. Cells were grown routinely in LB medium [38] at 37°C with shaking. Growth was initiated by inoculating fresh LB medium with 1∶100 dilutions of overnight cultures. Solid media contained 2% (w/v) agar, and plates were incubated overnight at 37°C.

Anaerobic growth (liquid and solid media) was carried out in a Coy chamber (Coy Laboratory Products, Inc.) under 85% N_2_, 10% H_2_, and 5% CO_2_. Anaerobic buffers and media were moved into the chamber immediately after being autoclaved and allowed to equilibrate with the anaerobic atmosphere for at least 24 h prior to use.

*E. coli* harboring pBAD or pBAD/*btuE* plasmids (see below) were grown in LB containing ampicillin (100 µg ml^−1^) at 37°C with continuous agitation. When the cultures reached an OD_600_ ∼0.4, L-arabinose (0.2% final concentration) was added. Induction was for 4 h at 37°C with shaking. Strains lacking *btuE* (Δ*btuE*) and all other mutants were grown in LB medium containing kanamycin (100 µg ml^−1^).

### Growth curves

To ensure that all studies were being conducted with exponentially growing cells, aerobic or anaerobic overnight cultures were diluted in fresh LB medium to an OD_600_ ∼0.005 and grown at 37°C until they achieved an OD_600_ of ∼0.1–0.2. Cultures were then diluted 10-fold into fresh medium containing K_2_TeO_3_ or H_2_O_2_, and they were grown at 37°C. Absorbance at 600 nm was monitored at 30 min intervals. Cell blackening due to tellurite reduction was negligible at tellurite concentrations up to 0.1 µg ml^−1^. In determining anaerobic growth, absorbance measurements were carried out at 1 h intervals.

### Cloning the *E. coli btuE* gene and strain construction

In order to amplify the *btuE* gene from the *E. coli* genome, specific primers (Table S1) were designed using the VECTOR 9 NTI (Invitrogen®) software. The PCR product was inserted into pBAD/TOPO (Invitrogen®) vector, according to manufacturer's instructions, resulting in plasmid pBAD/*btuE*. Identity/integrity of *btuE* was checked by DNA sequencing.

Strain Hpx^−^ Δ*btuE* was constructed by P1 transduction [39] between JEM216 x Δ*btuE* (Table S1), selecting for kanamicyn resistance. The *btuE* mutation in the resulting strain was confirmed by PCR using primers listed in Table S1.

### Determination of growth inhibition zones

Growth inhibition zones were determined in LB-agar plates as described [40]. In brief, overnight cultures were diluted with LB and grown at 37°C for 4 h. After dilution to an OD_600_ ∼0.1, 100 µl of each culture was evenly spread on the plates. Plates were air dried, and toxins to be tested (10 µl) were deposited on sterile 6 mm filter disks placed on the centres of the plates. Growth inhibition areas were determined after overnight incubation at 37°C. Determination of growth inhibition zones in anaerobic conditions followed an identical protocol, but all manipulations were carried out inside a Coy anaerobic chamber.

### Determination of the minimal inhibitory concentration

Sterile stock solutions of appropriate concentrations of K_2_TeO_3_, K_2_CrO_4_, CdCl_2_ or H_2_O_2_ were serially diluted in a 96-well ELISA plate containing 200 µl of LB medium (plus the appropriate antibiotic) per well. Five µl of cultures grown at 37°C in LB medium supplemented with the required antibiotic(s) to an OD_600_ ∼0.4 were added to each well, and the plate was incubated at 37°C. Turbidity was observed visually after 24 h. MIC determinations in anaerobic conditions followed the same protocol in a Coy chamber.

### Determination of intracellular reactive oxygen species

In general, cellular oxidants, including ROS, were assessed using the oxidation-sensitive probe 2′,7′-dichlorofluorescein diacetate. As demonstrated by Royall and Ischiropoulos [41], once inside the cell this esterified probe is deacetylated by intracellular esterases and the resulting compound, dichlorofluorescin, is susceptible to oxidation by ROS. Briefly, cells grown aerobically in LB medium to an OD_600_ ∼0.4 were exposed for 30 min to K_2_TeO_3_ (0.5 µg ml^−1^), paraquat (50 µg ml^−1^) or K_2_CrO_4_ (1 mM). They were then centrifuged, washed with 10 mM potassium phosphate buffer, pH 7.0, and incubated for 30 min in the same buffer containing the probe (10 mM final concentration). Cells were subsequently washed and disrupted by sonication. One hundred µl of the resulting cell extracts were mixed with 1 ml of the same buffer, and fluorescence intensity was determined using an Applied Biosystems Citofluor 4000 Fluorescence Multi-well plate reader (excitation 490 nm, emission 519 nm). Emission values were standardized by protein concentration [4], [42].

*E. coli* Δ*sodAB* and Hpx^−^ strains transformed with the indicated plasmids (Table S1) were used to determine intracellular ROS by flow cytometry. Cells were grown to an OD_600_ ∼0.5 in the presence of arabinose at 37°C, and they were then exposed to K_2_TeO_3_ (0.5 µg ml^−1^) for 30 min. After centrifugation at 5,000 *g* for 10 min, cells were washed with saline phosphate buffer (137 mM NaCl, 2.7 mM KCl, 10 mM Na_2_HPO_4_, 2 mM KH_2_PO_4_, pH 7.3) and diluted 1∶10 with the same buffer. Cells were incubated with 10 mM 2′,7′-dihydrodichlorofluorescein diacetate (or 127 µM dihydroethidine) for 30 min, centrifuged at 5,000 *g* for 10 min, and washed with the same buffer [43]. Fluorescence intensity was determined using a Becton Dickinson apparatus equipped with an argon laser.

### Determination of cytoplasmic protein oxidation

Oxidized cytoplasmic proteins were assessed as described by Semchyshyn *et al*. [27]. Briefly, nucleic acids-free cell extracts (100 µl) were prepared from cells exposed to K_2_TeO_3_ (0.5 µg ml^−1^) or H_2_O_2_ (100 µM) for 30 min. The extracts were mixed with 4 volumes of 10 mM 2,4-dinitrophenylhydrazine and incubated at room temperature for 1 h with occasional vortexing. Proteins were subsequently precipitated by the addition of 500 µl of 20% trichloroacetic acid, and precipitate was pelleted by centrifugation at 14,000 *g* for 5 min. After three washes with a 1∶1 solution of ethanol:ethyl acetate, the sediment was dissolved in 450 µl of 50 mM dithiothreitol in 6 M guanidine HCl at 37°C. Carbonyl content was determined spectrophotometrically at 370 nm using a molar absorption coefficient of 22,000 M^−1^cm^−1^ [4], [27].

### Determination of thiobarbituric acid-reactive substances

Cultures (4 ml) exposed or not exposed to K_2_TeO_3_ (0.5 µg ml^−1^) or H_2_O_2_ (100 µM) were centrifuged, washed twice, and suspended in 1 ml of 50 mM potassium phosphate buffer, pH 7.4, containing 0.1 mM butylated hydroxytoluene and 1 mM PMSF (phenylmethanesulfonyl fluoride). Cells were subjected to sonic disruption and centrifuged to discard the debris. The soluble fraction was mixed with 1 ml of 20% trichloroacetic acid and centrifuged at 10,000 *g* for 5 min. Supernatants were mixed with 2 ml of a saturated solution of thiobarbituric acid in 0.1 M HCl and 10 mM butylated hydroxytoluene. Samples were heated at 100°C for 1 h, and 1.5 ml aliquots were removed, cooled, mixed with 1.5 ml of butanol, and centrifuged at 4,000 *g* for 10 min. The organic fraction was removed, and the OD_535_ was determined. Thiobarbituric acid-reactive substances content was determined using an ε = 156 mM^−1^cm^−1^ [4], [27].

### Determination of membrane lipid peroxidation

The concentration of membrane lipid peroxides was determined as described by Cha *et al*. [16]. Briefly, 45 mg of cell sediment were suspended in Tris-HCl (pH 7.4) buffer containing 1% sodium dodecyl sulfate. After sonicating and washing with distilled water to remove the detergent, the sediment was air dried and dissolved in 1 ml of ethanol:chloroform (2∶1 v/v). After vigorous shaking for 1 h, FOXII reagent (ferrous oxidation in the presence of xylenol orange) was added, and the mixture was shaked again for 1 h at room temperature. After centrifuging at 13,000 *g* for 10 min, the clear supernatant was used to determine the content of membrane lipid peroxides at 560 nm [16].

## Supporting Information

Figure S1 **Effect of BtuE in the generation of intracellular ROS.** Cytoplasmic superoxide (**A**) or ROS (**B**) were determined by flow cytometry using dihydroethidine or 2′,7′-dihydrodichlorofluorescein diacetate in *E. coli* Δ*sodAB* or Hpx^−^ strains, respectively, exposed or not to K_2_TeO_3_ (0,5 µg/ml) for 30 min in the presence of 0.2% L-arabinose. Representative profiles of fluorescence intensity regarding the cell number (above) for the analyzed strains and histograms representing % of fluorescence intensity of control (pBAD) and pBAD/*btuE* cells (below) are shown. 100% of fluorescence intensity corresponds to the strain carrying pBAD only. Bars represent the average of three independent experiments ± SD. Numbers above each condition represent the pBAD/pBAD/*btuE* ratio.
(TIF)

Figure S2 **BtuE protects** ***E. coli*** **from potassium tellurite and hydrogen peroxide in aerobic conditions.** *E. coli* Hpx^−^ and Hpx^−^Δ*btuE* strains were grown aerobically (**A**) or anaerobically (**B**) in LB medium to an OD_600_ ∼0.01, and K_2_TeO_3_ was added to a final concentration of 0 (control, ○, •), 0.001 (□, ▪) and 0.005 µg ml^−1^ (Δ, ▴). Data are representative of three independent experiments. (**C**), Growth inhibition zones were assessed for Hpx^−^ and Hpx^−^ Δ*btuE* cells grown aerobically (+O_2_) or anaerobically (−O_2_) and exposed to K_2_TeO_3_ (10 µl, 1 µg/µl). Values represent the mean of three independent experiments ± SD. *E. coli* Hpx^−^ and Hpx^−^Δ*btuE* were grown aerobically (**D**) or anaerobically (**E**) in LB medium to an OD_600_ ∼0.01, and H_2_O_2_ was added to a final concentration of 0 (control, ○, •), 15 (□, ▪) and 30 µM (Δ, ▴). Data are representative of three independent experiments. (**F**), Growth inhibition zones were assessed for Hpx^−^ and Hpx^−^ Δ*btuE* cells grown aerobically (+O_2_) or anaerobically (−O_2_) and exposed to H_2_O_2_ (10 µl, 1 M). Values represent the mean of three independent experiments ± SD.
(TIF)

Table S1 **Bacterial strains, plasmids and primers used in this study.**
(DOCX)

Table S2 **BtuE mediates resistance to potassium tellurite and other ROS elicitors in** ***E. coli*** **.**
(DOCX)

Table S3 **Elimination of** ***btuE*** **results in decreased lipid peroxide levels in** ***E. coli*** **.**
(DOCX)
