# Supplementary figures and images for: The Escherichia coli BtuE Protein Functions as a Resistance Determinant against Reactive Oxygen Species
Source: PLoS One. 2011 Jan 10;6(1):e15979. doi: 10.1371/journal.pone.0015979 (PMC3018469; doi:10.1371/journal.pone.0015979)

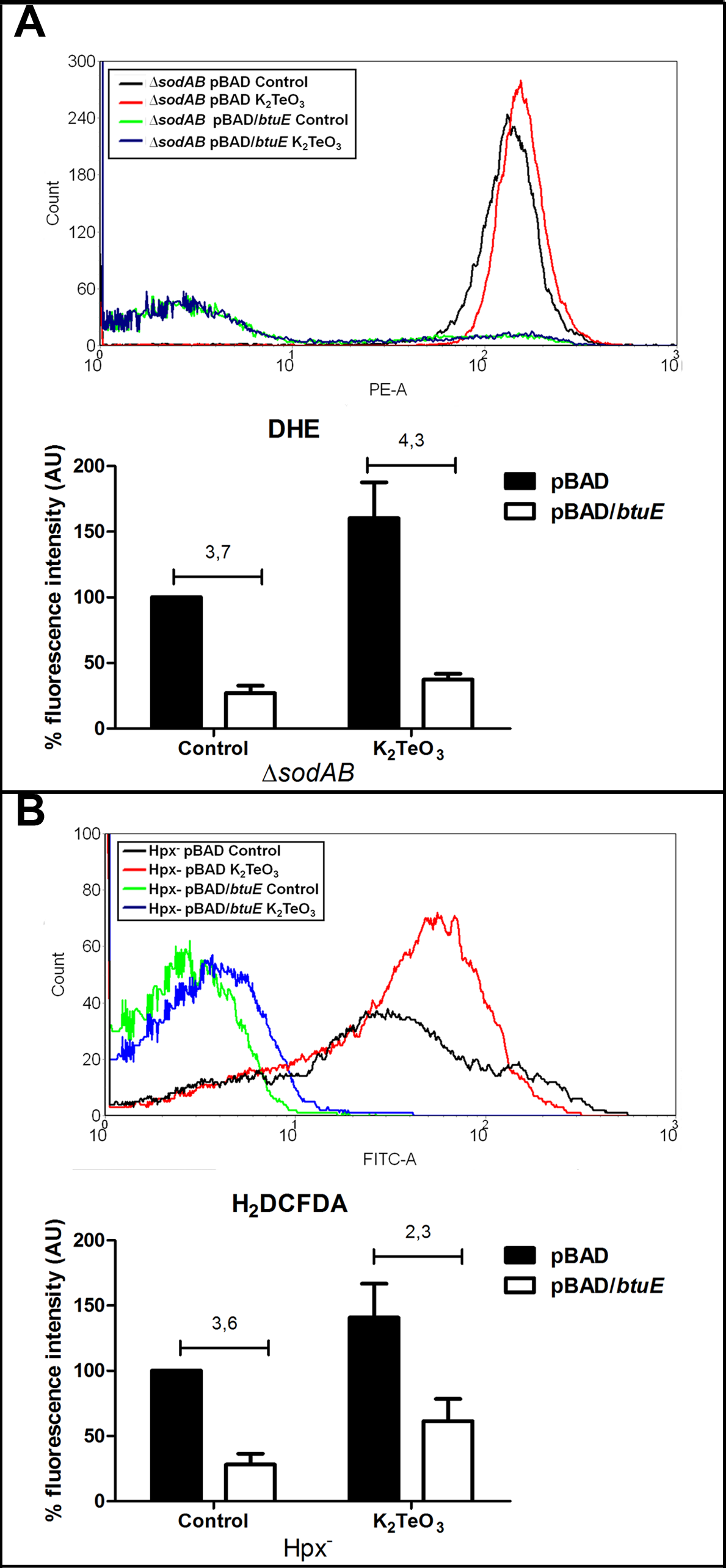

Supplement: Figure S1 — Effect of BtuE in the generation of intracellular ROS. Cytoplasmic superoxide (A) or ROS (B) were determined by flow cytometry using dihydroethidine or 2′,7′-dihydrodichlorofluorescein diacetate in E. coli ΔsodAB or Hpx− strains, respectively, exposed or not to K2TeO3 (0,5 µg/ml) for 30 min in the presence of 0.2% L-arabinose. Representative profiles of fluorescence intensity regarding the cell number (above) for the analyzed strains and histograms representing % of fluorescence intensity of control (pBAD) and pBAD/btuE cells (below) are shown. 100% of fluorescence intensity corresponds to the strain carrying pBAD only. Bars represent the average of three independent experiments ± SD. Numbers above each condition represent the pBAD/pBAD/btuE ratio. (TIF) [file pone.0015979.s001.tif]

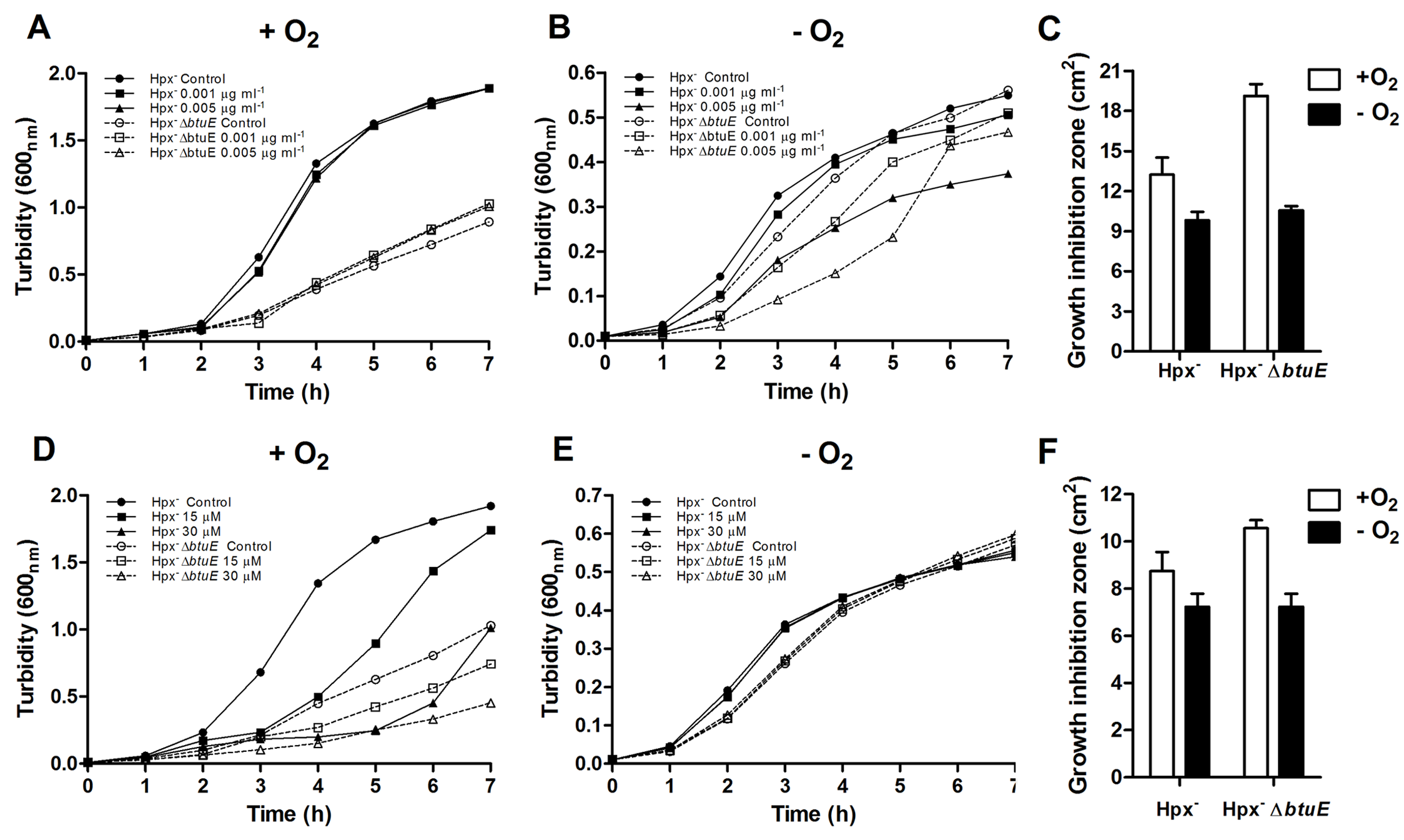

Supplement: Figure S2 — BtuE protects E. coli from potassium tellurite and hydrogen peroxide in aerobic conditions. E. coli Hpx− and Hpx−ΔbtuE strains were grown aerobically (A) or anaerobically (B) in LB medium to an OD600 ∼0.01, and K2TeO3 was added to a final concentration of 0 (control, ○, •), 0.001 (□, ▪) and 0.005 µg ml−1 (Δ, ▴). Data are representative of three independent experiments. (C), Growth inhibition zones were assessed for Hpx− and Hpx− ΔbtuE cells grown aerobically (+O2) or anaerobically (−O2) and exposed to K2TeO3 (10 µl, 1 µg/µl). Values represent the mean of three independent experiments ± SD. E. coli Hpx− and Hpx−ΔbtuE were grown aerobically (D) or anaerobically (E) in LB medium to an OD600 ∼0.01, and H2O2 was added to a final concentration of 0 (control, ○, •), 15 (□, ▪) and 30 µM (Δ, ▴). Data are representative of three independent experiments. (F), Growth inhibition zones were assessed for Hpx− and Hpx− ΔbtuE cells grown aerobically (+O2) or anaerobically (−O2) and exposed to H2O2 (10 µl, 1 M). Values represent the mean of three independent experiments ± SD. (TIF) [file pone.0015979.s002.tif]
