# Supplementary material for: The Escherichia coli BtuE Protein Functions as a Resistance Determinant against Reactive Oxygen Species
Source: PLoS One. 2011 Jan 10;6(1):e15979. doi: 10.1371/journal.pone.0015979 (PMC3018469; doi:10.1371/journal.pone.0015979)
Supplement: Table S1 — Bacterial strains, plasmids and primers used in this study. (DOCX) [file pone.0015979.s003.docx]

**Table S1.** Bacterial strains, plasmids and primers used in this study

| ***E. coli* strain** | **Relevant genotype** | **Source or reference** |
| --- | --- | --- |
| BW25113 | Wild-type, *lacI*^q^ *rrnB*_T14_ ∆*lacZ*_WJ16_ *hsdR*514 ∆*araBAD*_AH33_ ∆*rhaBAD*_LD78_ | [19] |
| BW25113 pBAD | BW25113 harboring pBAD | This work |
| BW25113 pBAD/*btuE* | BW25113 harboring pBAD/*btuE* | This work |
| ∆*btuE* | BW25113 ∆*btuE (btuE::kan*) | NARA Institute, Japan |
| ∆*btuE* pBAD | BW25113 ∆*btuE* harboring pBAD | This work |
| ∆*btuE* pBAD/*btuE* | BW25113 ∆*btuE* harboring pBAD/*btuE* | This work |
| Δ*katE* | BW25113 ∆*katE (katE::kan)* | NARA Institute, Japan |
| Δ*katE* pBAD | BW25113 ∆*katE* harboring pBAD | This work |
| Δ*katE* pBAD/*btuE* | BW25113 ∆*katE* harboring pBAD/*btuE* | This work |
| Δ*katG* | BW25113 ∆*katG (katG::kan)* | NARA Institute, Japan |
| Δ*katG* pBAD | BW25113 ∆*katG* harboring pBAD | This work |
| Δ*katG* pBAD/*btuE* | BW25113 ∆*katG* harboring pBAD/*btuE* | This work |
| Δ*ahpC* | BW25113 ∆*ahpC (ahpC::kan)* | NARA Institute, Japan |
| Δ*ahpC* pBAD | BW25113 ∆*ahpC* harboring pBAD | This work |
| Δ*ahpC* pBAD/*btuE* | BW25113 ∆*ahpC* harboring pBAD/*btuE* | This work |
| Δ*ahpF* | BW25113 ∆*ahpF (ahpF::kan)* | NARA Institute, Japan |
| Δ*ahpF* pBAD | BW25113 ∆*ahpF* harboring pBAD | This work |
| Δ*ahpF* pBAD/*btuE* | BW25113 ∆*ahpF* harboring pBAD/*btuE* | This work |
| JEM216 | ∆(*ahpCF1*::*cam*)1 ∆*katG17*::Tn10 ∆(*katE12*::Tn10)1, Kan^S^ | James Imlay |
| Hpx^-^  ∆*btuE* | ∆*btuE*::*kan* Δ*ahpCF*´ *kan*::’*ahpF* Δ(*katG17*::Tn*10*)1 Δ(*katE12*::Tn*10*)1 (P1 JEM216 X ∆*btuE*) | This work |
| MG1655 | Wild-type, F^-^ lambda^-^ *ilvG*^-^ *rfb*-50 *rph*^-^1 | James Imlay |
| MG1655 pBAD | MG1655 harboring pBAD | This work |
| MG1655 pBAD/*btuE* | MG1655 harboring pBAD/*btuE* | This work |
| Hpx^-^ | LC106 Δ*ahpCF*´ *kan*::’*ahpF* Δ(*katG17*::Tn*10*)1 Δ(*katE12*::Tn*10*)1 | James Imlay |
| Hpx^-^ pBAD | LC106 harboring pBAD | This work |
| Hpx^-^ pBAD/*btuE* | LC106 harboring pBAD *btuE* | This work |
| MP *btuE* | JA200 harboring pNT3/*btuE.* Amp^R^ | NARA Institute, Japan |
| MP *katG* | JA200 harboring pNT3/*katG.* Amp^R^ | NARA Institute, Japan |
| MP *katE* | JA200 harboring pNT3/*katE.* Amp^R^ | NARA Institute, Japan |
| MP *bcp* | JA200 harboring pNT3/*bcp.* Amp^R^ | NARA Institute, Japan |
| MP *txp* | JA200 harboring pNT3/*tpx.* Amp^R^ | NARA Institute, Japan |
| JA200 | silvestre, F+ *thr-1*, *leu-6*, DE*(trpE)5*, *recA*, *lacY*, *thi*, *gal*, *xyl*, *ara*, *mtl*). | NARA Institute, Japan |
| ADA110 | *AB734 λΦ (ibp::lacZ)* | Shapiro y Baneyx , 2002 [28] |
| ADA110 pBAD | ADA110 harboring pBAD. Amp^R^ | This work |
| ADA110 pBAD/*btuE* | ADA110 harboring pBAD/*btuE*. Amp^R^ | This work |
| SP11 | Δ*lac*U169, *rpsL*, λ (*soxS*::*lacZ*) | James Imlay |
| SP11 pBAD | SP11 harboring pBAD. Amp^R^ | This work |
| SP11 pBAD/*btuE* | SP11 harboring pBAD/*btuE*. Amp^R^ | This work |
| GS022 | *ara*D139 DE(*arg*F-*lac*)169 λ^-^*flh*D5301 *fru*A25 *rel*A1 *rps*L150(Str^R^) *rbs*R22 *deo*C1 λ RS45 (*katG*::*lacZ*) | James Imlay |
| GS022 pBAD | GS022 harboring pBAD. Amp^R^ | This work |
| GS022 pBAD/*btuE* | GS022 harboring pBAD/*btuE*. Amp^R^ | This work |
| Δ*sodAB* | DE(*lac*)4169 *rps*L DE(*sodA*-*lacZ*)49 DE(*sod*B-*kan*)1-DE(2) Cam^R^ Kan^R^ | James Imlay |
| Δ*sodAB* pBAD | Δ*sodAB* harboring pBAD. Cam^R^ Kan^R^ Amp^R^ | This work |
| Δ*sodAB* pBAD/*btuE* | Δ*sodAB* harboring pBAD/*btuE*. Cam^R^ Kan^R^ Amp^R^ | This work |
| **Plasmids** | **Relevant characteristics** | **Source or reference** |
| pBAD TOPO | Expression vector, Ap^R^ | INVITROGEN^®^ |
| pBAD/*btuE* | pBAD harboring the *E.coli* *btuE* gene, Ap^R^ | This work |
| pCP20 | encodes *flp*, Ap^R^, t^s^ | James Imlay |
| **Primers** | **Forward (F) and reverse (R) primers to** | **5’- 3’ Sequence** |
| *btuE*F | PCR *btuE*, forward | ATGCAAAACTCGCTTCTTAATACTC |
| *btuE*R | PCR *btuE*, reverse | TTTTGCCAACGCCAGTTTAATGC |
| *btuE*P1F | Check *btuE* elimination, forward | GAT TTT TAC TGA CCA CAC CCA G |
| *btuE*P1R | Check *btuE* elimination , reverse | CAA CAT CTT GTA ACT GCA TCA CA |
| *kan*F | PCR *kan*, forward | ATGATTGAACAAGATGGATTGC |
| *kan*R | PCR *kan*, reverse | ACCATGATATTCGGCAAGCA |
