# Supplementary material for: The Escherichia coli BtuE Protein Functions as a Resistance Determinant against Reactive Oxygen Species
Source: PLoS One. 2011 Jan 10;6(1):e15979. doi: 10.1371/journal.pone.0015979 (PMC3018469; doi:10.1371/journal.pone.0015979)
Supplement: Table S2 — BtuE mediates resistance to potassium tellurite and other ROS elicitors in E. coli . (DOCX) [file pone.0015979.s004.docx]

**Table S2.** BtuE mediates resistance to potassium tellurite and other ROS elicitors in *E. coli*.

**Toxicant**

| **Strain** | **K_2_TeO_3_** | **H_2_O_2_** | **K_2_CrO_4_** | **CdCl_2_** |
| --- | --- | --- | --- | --- |
| **BW25113 pBAD** | 4 | 3,300 | 700 | 1,600 |
| **BW25113 pBAD/*btuE*** | 17 | 30,000 | 2,600 | 1,600 |
| **∆*btuE* pBAD** | 2 | 3,700 | 900 | 1,200 |
| **∆*btuE* pBAD/*btuE*** | 6 | 19,300 | 2,300 | 1,200 |

Minimal inhibitory concentrations (μM) of K_2_TeO_3_, K_2_CrO_4_, H_2_O_2_ and CdCl_2_ were determined initially using serial dilutions as described in Methods. Values (the mean of three independent determinations) were further delimited using sterile solutions of appropriate concentrations of these compounds.
