# Supplementary material for: The Escherichia coli BtuE Protein Functions as a Resistance Determinant against Reactive Oxygen Species
Source: PLoS One. 2011 Jan 10;6(1):e15979. doi: 10.1371/journal.pone.0015979 (PMC3018469; doi:10.1371/journal.pone.0015979)
Supplement: Table S3 — Elimination of btuE results in decreased lipid peroxide levels in E. coli . (DOCX) [file pone.0015979.s005.docx]

**Table S3.** Elimination of *btuE* results in decreased lipid peroxide levels in *E. coli*.

A_560_/mg protein

| Strain | Control | K_2_TeO_3_ | H_2_O_2_ |
| --- | --- | --- | --- |
| BW25113 pBAD | 0.060 ± 0.003 | 0.067 ± 0.004 | 0.068 ± 0.004 |
| BW25113 pBAD/*btuE* | 0.047 ± 0.003 | 0.058 ± 0.003 | 0.052 ± 0.002 |
| Δ*btuE* pBAD | 0.119 ± 0.006 | 0.112 ± 0.008 | 0.111 ± 0.005 |
| Δ*btuE* pBAD/*btuE* | 0.061 ± 0.004 | 0.061 ± 0.003 | 0.056 ± 0.006 |

Lipid peroxides were determined using the indicated *E. coli* strains grown in the presence of arabinose (0.2 %) and exposed or not exposed to K_2_TeO_3_ (0.5 μg/ml) or H_2_O_2_ (100 μM) for 20 min. See Methods for further details. Values are the mean of 3 independent trials ± SD.
